# Supplementary material for: Three Novel Cheiroid Hyphomycetes in Dictyocheirospora and Dictyosporium (Dictyosporiaceae) from Freshwater Habitats in Guangdong and Guizhou Provinces, China
Source: J Fungi (Basel). 2024 Mar 28;10(4):259. doi: 10.3390/jof10040259 (PMC11051510; doi:10.3390/jof10040259)
Supplement: Supplementary file 1 [file jof-10-00259-s001.zip › jof-2904433-supplementary.pdf]

Journal of *Fungi*

MDPI

Article

Three novel cheiroid hyphomycetes in *Dictyocheirospora* and *Dictyosporium* (Dictyosporiaceae) from freshwater habitats in Guangdong and Guizhou Provinces, China

Yongxin Shu [1,2,3], Mingkwan Doilom [1], Saranyaphat Boonmee [2,3], Biao Xu [1], and Wei Dong [1,*]

1 Innovative Institute for Plant Health / Key Laboratory of Green Prevention and Control on Fruits and Vegetables in South China, Ministry of Agriculture and Rural Affairs, Zhongkai University of Agriculture and Engineering, Guangzhou 510225, Guangdong, China
2 Center of Excellence in Fungal Research, Mae Fah Luang University, Chiang Rai 57100, Thailand
3 School of Science, Mae Fah Luang University, Chiang Rai 57100, Thailand
* Correspondence: dongwei0312@hotmail.com

Table S1. Taxa used for phylogenetic analyses and their corresponding GenBank accession numbers. Newly generated sequences are indicated in red, ex-type strains are indicated in bold, and missing sequences are indicated with "–".

Species	Source	GenBank accession number			
		LSU	SSU	ITS	*tef1-α*
Aquadictyospora clematidis	**MFLUCC 17-2080**	**MT214545**	**MT226664**	**MT310592**	**MT394727**
Aquadictyospora lignicola	**MFLUCC 17-1318**	**MF948629**	**–**	**MF948621**	**MF953164**
Cheirosporium triseriale	**HMAS 180703**	**EU413954**	**–**	**EU413953**	**–**
Dendryphiella phitsanulokensis	**MFLUCC 17-2513**	**MG754401**	**MG754402**	**MG754400**	**–**
Dendryphiella variabilis	**CBS 584.96**	**LT963454**	–	**LT963453**	–
Dictyocheirospora acaciae	SDBR-CMU456	OP965374	–	OP965334	OQ000840
Dictyocheirospora acaciae	SDBR-CMU455	OP965373	–	OP965332	OQ000839
Dictyocheirospora aquadulcis	**MFLUCC 17-2571**	**MK634542**	**–**	**MK634545**	**–**
Dictyocheirospora aquatica	**KUMCC 15-0305**	**KY320513**	**–**	**KY320508**	**–**
Dictyocheirospora bannica	**KH 332**	**AB807513**	**AB797223**	**LC014543**	**AB808489**
Dictyocheirospora bannica	MFLUCC 16-0874	MH381774	–	MH381765	–
Dictyocheirospora cheirospora	**KUMCC 17-0035**	**MF177036**	**MF928073**	**MF177035**	**–**
Dictyocheirospora chiangmaiensis	**MFLUCC 22-0097**	**OP526640**	**OP526621**	**OP526630**	**OP542232**
Dictyocheirospora clematidis	**MFLUCC 17-2089**	**MT214546**	**MT226665**	**MT310593**	**MT394728**
Dictyocheirospora gareth-jonesii	**MFLUCC 16-0909**	**KY320514**	**–**	**KY320509**	**–**
Dictyocheirospora gareth-jonesii	DLUCC 0848	MF948631	–	MF948623	MF953166
Dictyocheirospora gigantica	BCC 11346	–	–	DQ018095	–
Dictyocheirospora heptaspora	CBS 396.59	–	DQ018082	DQ018090	–
Dictyocheirospora indica	MFLUCC 15-0056	MH381772	MH381757	MH381763	MH388817
Dictyocheirospora lithocarpi	**MFLUCC 17-2537**	**MK347999**	**MK347888**	**MK347781**	**–**
Dictyocheirospora metroxylonis	**MFLUCC 15-0028a**	**MH742313**	**MH742317**	**MH742321**	–
Dictyocheirospora metroxylonis	**MFLUCC 15-0028b**	**MH742314**	**MH742318**	**MH742322**	**MH764301**
Dictyocheirospora multiappendiculata	KUNCC 22-10736	OP526643	OP526624	OP526633	OP542235
Dictyocheirospora multiappendiculata	**KUNCC 22-10734**	**OP526642**	**OP526623**	**OP526632**	**OP542234**

Species	Source	GenBank accession number			
		LSU	SSU	ITS	*tef1-α*
Dictyocheirospora nabanheensis	**MFLUCC 17-0562**	**MH376712**	–	**MH388340**	**MH388375**
Dictyocheirospora nabanheensis	MFLU 19-0538	MN511737	–	MN513035	–
Dictyocheirospora nabanheensis	C317	MK347973	MK347862	MK347756	–
Dictyocheirospora pandanicola	**MFLUCC 16-0365**	**MH376713**	**MH388309**	**MH388341**	**MH388376**
Dictyocheirospora pseudomusae	**yone 234**	**AB807520**	**AB797230**	**LC014550**	**AB808496**
Dictyocheirospora rotunda	**MFLUCC 14-0293**	**KU179100**	**KU179101**	**KU179099**	–
Dictyocheirospora rotunda	MFLUCC 17-0222	MH381773	–	MH381764	MH388818
Dictyocheirospora suae	**KUNCC 22-12424**	**OP526641**	**OP526622**	**OP526631**	**OP542233**
Dictyocheirospora submersa	**MHZU 24-0282**	**PP326216**	**PP335106**	**PP326193**	**PP333133**
Dictyocheirospora subramanianii	BCC 3503	–	–	DQ018094	–
Dictyocheirospora taiwanense	**MFLUCC 17-2654**	**MK495820**	–	**MK495821**	–
Dictyocheirospora thailandica	**MFLUCC 18-0987**	**MN913743**	–	**MT627734**	–
Dictyocheirospora vinaya	**MFLUCC 14-0294**	**KU179103**	**KU179104**	**KU179102**	–
Dictyocheirospora xishuangbannaensis	**KUMCC 17-0181**	**MH376714**	**MH388310**	**MH388342**	**MH388377**
Dictyocheirospora xishuangbannaensis	MFLUCC 17-2087	MT214547	MT226666	MT310594	MT394729
Dictyosporium alatum	**ATCC 34953**	**DQ018101**	**DQ018080**	**DQ018088**	–
Dictyosporium appendiculatum	**KUMCC 17-0311**	**MH376715**	–	**MH388343**	–
Dictyosporium aquaticum	**MF 1318**	–	–	**KM610236**	–
Dictyosporium bulbosum	yone 221	AB807511	AB797221	LC014544	AB808487
Dictyosporium digitatum	SDBR-CMU459	OP965377	–	OP965337	OQ000841
Dictyosporium digitatum	KUMCC17-0269	MH376716	MH388311	MH388344	–
Dictyosporium digitatum	KH401	AB807515	AB797225	LC014545	AB808491
Dictyosporium digitatum	KT2660	AB807518	AB797228	LC014546	AB808494
Dictyosporium digitatum	yone 208	AB807512	AB797222	LC014547	AB808488
Dictyosporium digitatum	MHZU 24-0285	PP326214	PP335104	PP326191	PP333111
Dictyosporium duliujiangense	GZCC 19-0426	MW133815	–	OQ842725	OQ850746
Dictyosporium elegans	**NBRC 32502**	**DQ018100**	**DQ018079**	**DQ018087**	–
Dictyosporium guangdongense	**MHZU 24-0283**	**PP326213**	**PP335103**	**PP326190**	–
Dictyosporium guttulatum	**MFLUCC 16-0258**	**MH376717**	**MH388312**	**MH388345**	**MH388379**
Dictyosporium hongkongensis	**KMUCC 17-0268**	**MH376718**	**MH388313**	**MH388346**	**MH388380**
Dictyosporium hughesii	**KT 1847**	**AB807517**	**AB797227**	**LC014548**	**AB808493**
Dictyosporium karsti	**MFLU 18-2282**	–	**OR134404**	**OR225025**	**OR140390**
Dictyosporium krabiense	**MFLU 16-1890**	**MH376719**	**MH388314**	–	**MH388381**
Dictyosporium marinum	**GJ357**	**MN017841**	–	–	–
Dictyosporium meiosporum	**MFLUCC 10-0131**	**KP710945**	**KP710946**	**KP710944**	–
Dictyosporium muriformis	**GZCC 20-0006**	**MN897834**	**MN901117**	**MT002304**	**MT023011**
Dictyosporium nigroapice	MFLUCC 17-2053	MH381777	MH381762	MH381768	MH388821
Dictyosporium nigroapice	BCC 3555	–	–	DQ018085	–
Dictyosporium olivaceosporum	**KH 375**	**AB807514**	**AB797224**	**LC014542**	**AB808490**
Dictyosporium palmae	**CBS-H 22129**	**KX555648**	–	–	–
Dictyosporium pandanicola	**MFLU 16-1886**	**MH376720**	–	**MH388347**	**MH388382**
Dictyosporium sexualis	**MFLUCC 10-0127**	**KU179106**	**KU179107**	**KU179105**	–
Dictyosporium sp.	**MFLUCC 15-0629**	MH381775	–	MH381766	MH388819
Dictyosporium stellatum	**CCFC 241241**	**JF951177**	–	**NR_154608**	–
Dictyosporium strelitziae	**CBS 123359**	**FJ839653**	–	**NR_156216**	–
Dictyosporium tetrasporum	**KT 2865**	**AB807519**	**AB797229**	**LC014551**	**AB808495**

Species	Source	GenBank accession number			
		LSU	SSU	ITS	*tef1-α*
Dictyosporium thailandicum	**MFLUCC 13-0773**	**KP716707**	**–**	**KP716706**	**–**
Dictyosporium tratense	**MFLUCC 17-2052**	**MH381776**	**MH381761**	**MH381767**	**MH388820**
Dictyosporium tubulatum	**MFLUCC 15-0631**	**MH381778**	**–**	**MH381769**	**MH388822**
Dictyosporium tubulatum	MFLUCC 17-2056	MH381779	–	MH381770	–
Dictyosporium variabilisporum	**MHZU 24-0284**	**PP326215**	**PP335105**	**PP326192**	**PP333112**
Dictyosporium wuyiense	**CGMCC 3.18703**	**–**	**–**	**KY072977**	**–**
Dictyosporium zhejiangense	**MW-2009a**	**–**	**–**	**FJ456893**	**–**
Digitodesmium chiangmaiense	**KUN-HKAS 102163**	**MK571766**	**MK571775**	**–**	**–**
Digitodesmium polybrachiatum	**COAD 3174**	**MW879316**	**MW879325**	**MW879318**	–
Digitodesmium polybrachiatum	COAD 3175	MW879317	MW879326	MW879319	–
Gregarithecium curvisporum	KT 922	AB807547	AB797257	AB809644	–
Gregarithecium curvisporum	**MFLUCC 13-0853**	**KX364282**	**KX364283**	**KX364281**	**–**
Immotthia atrograna	ZT-Myc-64283	–	–	MW489540	–
Immotthia bambusae	**KUN-HKAS 112012A1**	**MW489450**	**MW489461**	**MW489455**	**MW504646**
Jalapriya inflata	NOU 3855	JQ267363	JQ267361	JQ267362	–
Jalapriya pulchra	**MFLUCC 15-0348**	**KU179109**	**KU179110**	**KU179108**	**–**
Neodendryphiella mali	**FMR 16561**	LT906657	–	LT906655	–
Neodendryphiella tarraconensis	**GZCC 20-0002**	MN999927	–	MN999922	–
Periconia igniaria	CBS 379.86	AB807566	–	LC014585	AB808542
Periconia igniaria	CBS 845.96	AB807567	–	LC014586	AB808543
Pseudocoleophoma bauhiniae	**MFLUCC 17-2586**	**MK347953**	**MK347844**	**MK347736**	**MK360076**
Pseudocoleophoma calamagrostidis	**KT 3284**	**LC014609**	**LC014604**	**LC014592**	**LC014614**
Pseudocoleophoma flavescen	CBS 178.93	GU238075	GU238216	–	–
Pseudocoleophoma zingiberacearum	**NCYUCC 19-0052**	**MN616753**	–	**MN615939**	**MN629281**
Pseudocyclothyriella clematidis	**MFLUCC 17-2177**	**MT214549**	–	**MT310596**	**MT394730**
Pseudocyclothyriella clematidis	**MFLUCC 17-2177A**	**MT214548**	**MT226667**	**MT310595**	**–**
Pseudodictyosporium elegans	**CBS 688.93**	**DQ018106**	**DQ018084**	**DQ018099**	**–**
Pseudodictyosporium wauense	NBRC 30078	DQ018105	DQ018083	DQ018098	–
Pseudodictyosporium wauense	DLUCC 0801	MF948630	–	MF948622	MF953165
Verrucoccum coppinsii	**E00396112**	**MT918766**	**MT918774**	**MT918782**	–
Verrucoccum spribillei	**SPO1154**	**MT918764**	**MT918772**	**MT918781**	–
Vikalpa australiensis	HKUCC 8797	–	–	DQ018092	–
Vikalpa grandispora	**KUNCC 22-12425**	**OP526648**	**OP526628**	**OP526638**	**OP542240**
Vikalpa sphaerica	CGMCC 3.20682	OP526649	OP526629	OP526639	OP542A85
